# Progesterone in the Treatment of Advanced Malignant Tumours of Breast, Ovary and Uterus*

**DOI:** 10.1038/bjc.1962.24

**Published:** 1962-06

**Authors:** B. Jolles


### PROGESTERONE IN THE TREATMENT OF ADVANCED MALIGNANT TUMOURS OF BREAST, OVARY AND UTERUS*

B. JOLLES

*From the Department of Radiotherapy, General Hospital, Northampton*

Received for publication April 27, 1962

WHILE there is a vast literature on the effects of oestrogens and androgens on tumours, only a few reports have appeared in the last two or three years on clinical experience with progestins in human cancer. Kelley and Baker (1961). have treated 21 cases of uterine cancer using various preparations of progestational agents. Stoll (1961) treated 2 patients with oral progestational steroid (Enavid) and noted a regression of their recurrent endometrial carcinomata. No reports on cases of malignant ovarian tumours and very few on breast cancer treated with progesterone were found in the literature. Twelve cases of advanced mammary carcinoma were treated by Douglas, Loraine and Strong (1960) who studied the effects of 19-norethistosterone oenanthate, an early non-marketed German product. Only one patient in their series showed a favourable response. The indications for the use of a hormone which appears to exert its biological activity through an influence on connective tissue structures need hardly be stressed. The availability of a long acting progesterone preparation in the form of Primolut Depot (Delalutin in U.S.A.) prompted, in 1957, the start of a study of its growth-restraining effects in a small series of cases of advanced malignant tumours of breast, ovary and uterus. The findings and the discussion of the rationale of treatment are the subject of this preliminary note.

### THE HORMONE AND CLINICAL MATERIAL

It is generally accepted that the pharmacological properties of synthetic progesterone are comparable to natural corpus luteum hormone. The disadvantage of its low solubility, hence underdosage in clinical use, has been overcome by the development of hydroxyprogesterone capronate by hydrolysis from progesterone. Concentrated in Primolut Depot up to 250 mg. in 1 ml. of oily solution it has an activity lasting over one week. Other derivatives such as 17$\alpha$-alkyl-19-nor-testosterone (Junkmann, 1954) with the trade name of Primolut N and 17$\alpha$-ethinyl-17-hydroxy-5(10)-oestren-3-one, enhanced by ethinyloestradiol 3-methyl ether, marketed as Enavid, have the advantage of a marked oral activity. The capronate form has a progestational action, prompt and twice as marked and 2 to 4 times more prolonged than free progesterone. Kistner (1958) claims that Primolut Depot in comparison with free 17$\alpha$-hydroxyprogesterone is 30 times more effective and 5 times more prolonged in its action. It is claimed to have a weak anti-gonadotrophic action and, similar to progesterone, little effect on the menstrual cycle. It has shown no androgenic effects in animal experiments (Suchowsky and Junkmann, 1961).

* Based on a paper read at the 2nd Annual Conference of the British Association of Cancer Research held at the Middlesex Hospital Medical School, London, on September 16, 1961.

TABLE II.—*Carcinoma Breast*

| Case (Age) | Histology | Surgery | Radiotherapy | Recurrence (R) or Metastases (M) | Oestrogens or Androgens | Primolut | Improvement | Survival |
|---|---|---|---|---|---|---|---|---|
| (1) A. M— (46) | Spheroidal cell adenocarcinoma with scirrhous areas | 27.vi.57. Local mastectomy. 8.vii.57. Bilateral salpingo-oophorectomy | 29.vii.57. DXR to breast and hip | vii.57. Pelvis and left femoral head | vii.57. Two injections of testosterone 200 mg. each 25.x.57 to 28.i.59 weekly Durabolin (25 mg.) injections | 4.iv.59. 30 mg. Primolut N. | Good | 26 months from surgery 6 months from Primolut. |
| (2) S. P— (46) | Scirrhous carcinoma. Lymphatic secondaries | 19.vi.53. Rad. mastectomy | 16.vii.53. DXR | | | | Good throughout | Still alive. |
| | vii.57. Intestinal metastases from carcinoma breast | 5.vii.57. Resection of ileum with mesentric glands. Histology +++ | | vii.57. Intestinal metastases. 11.ix.57. Skin nodules (M). 1958. Mass in opposite breast | | 11.ix.57 to 28.ii.62 232 injections (250 mg.) =58,000 mg. Continuing | | |
| (3) B. A— (55) | Carcinoma implex. Lymph nodes involved | 1.xii.56. Rad. mastectomy | 17.xii.56 DXR. 10.xi.59 DXR to M. 15.iii.61 DXR to hip and lumbo-sacral region. 26.vi.61 DXR to cervical spine | xii.58. Lumbar spine (M) xi.1959. Bone metastases in femur, lumbar and dorsal spine | xi.59. Durabolin 4 injections | xii.59. 13 × 250 mg. = 3,250 mg. | Slight improvement for 2 months | 30 months from surgery. 7 months from Primolut. |
| (4) G. F— (67) | Cellular poorly diff. carcinoma. Lymph nodes involved | 27.i.49. Radical mastectomy | 28.iv.49 DXR to axilla and s/clavicular fossa. 18.viii.50. Radium moulds to infra-clavicular, para-sternal and axillary (R) | 22.ii.61. Sloughy necrotic 3 × 2 cm area in lt. infra-clavicular region (R) | | Between 21.iv.61 and ix.61. 9 injections. 17.i.62 further injections prescribed. Total: 15 × 250 mg. = 3,750 mg. | Good throughout. Ulcerated area almost completely healed | Still alive and well. |

| Case (Age) | Histology | Surgery | Radiotherapy | Reccurrence (R) or Metastases (M) | Oestrogens or Androgens | Primolut | Improvement | Survival |
|---|---|---|---|---|---|---|---|---|
| (5) S. H— (66) | Carcinoma simplex | 29.ii.52. Radical mastectomy | | iv.58. Widespread bone metastases | 6.v.58. 200 mg. testosterone | 9.vi.58. 3 ampoules 250 mg. ea. =750 mg. | Nil | 1 month from Primolut. |
| (6) C. S— (52) | Carcinoma simplex. No lymph nodes | 26.iv.60. Radical mastectomy | 27.iii.61 DXR to recurrence in rt. breast area and mass in lt. breast | ii.1961. Rt. breast (R). vi.61. Rt. side of pelvis and hip (M) | vi.61. 3 injections of Durabolin | 17.iv.61. 6 injections. Total : 6 × 250 mg. = 1,500 mg. | Nil | 16 months from surgery. 4 months DXR. 3 months from Primolut. |
| 7) S. P— (41) | Spheroidal cell carcinoma | 4.ix.58. Local mastectomy | 22.x.58 DXR to lt. iliac region | 15.x.58. Lt. iliac region (M) | 20.x.58. Durabolin injections prescribed | iii.59. Injections twice weekly. 60 × 250 mg. = 15,000 mg. | Fair for 3 months | 14 months from surgery. 13 months DXR. 8 months from Primolut. |
| (8) D. W— (54) | | 23.v.60. Local mastectomy. 21.xi.60. Bilateral salpingo-oophorectomy | 5.x.59 DXR aiming at growth restraint. 10.i.61 to 23.i.61 6 injections of Trillekamine 6 mg. each, i.v. | 2.xi.60. Glands in lt. upper neck (M). Widespread infiltration of clavicular region. Mediastinal mass | x.59. Dienoestrol 10 mg. *t.d.s.* | 7.iv.61. Primolut commenced | Nil | 20 months from DXR. 2 months from Primolut. |
| | | *Post mortem report :* | 1(*a*) Pulmonary embolus, due to 1(*b*) metastases from carcinoma of left breast. | | | | | |
| | | *Carcinoma of breast:* | Section shows invasion of mediastinum and liver capsule by well differentiated scirrhous carcinoma of breast. | | | | | |
| (9) D. M— (61) | Nil | 28.v.59. Local mastectomy | 27.v.59 DXR to sacro-lumbar region (M) | | 10.vi.59. Durabolin injections commenced | 10.viii.59. Primolut commenced | Nil | 3 months from surgery and DXR. ? 2 weeks from Primolut. |

### Table III.—*Carcinoma Ovary*

| Case (Age) | Histology | Surgery | Radiotherapy or Chemotherapy | Recurrence (R) or Metastases (M) | Primolut | Improvement | Survival |
|---|---|---|---|---|---|---|---|
| (10) M. E— (72) | Nil | Nil<br>Seen at Royal Bucks Hospital, Aylesbury in May, 1957. From May to September, 1957, had monthly paracenteses. | Nil | | ix.57 to iv.58 24 24 × 250 mg. injections (6,000 mg.) | Good for 6 months. No ascites | 13 months from Primolut. |
| (11) C. E— (57) | Poorly differentiated adenocarcinoma | 26.iv.57. Laparotomy | 17.v.57 to 21.vi.57 DXR to pelvis and abdomen | 26.iv.57. Noted at laparotomy : secondaries dotted over liver and intestines | i.58. 4 × 250 mg. injections (1,000 mg.) | None | 9 months from R/therapy.<br>1 month from Primolut. |
| (12) O. E— (43) | Moderately differentiated papillary adenocarcinoma | 19.i.57. Total hysterectomy and bilateral salpingo-oophorectomy | 11.ii.57 to 5.iii.57 DXR | 19.vi.61. Mobile mass above Poupart's ligament (M) | From 19.vi.61. 15 × 250 mg. injections (3,700 mg.), continuing | Good reduction of size of tumour | Still alive and well. |
| (13) F. B— (38) | Poorly differentiated psammonatous adenocarcinoma | 20.i.61. Laparotomy | i.61. Nitrogen mustard 5 × 15 mg. | 20.i.61. Metastases in the pelvic peritoneum and serosal surface of the pelvic colon | From i.61. 74 × 250 mg. injections (18,500 mg.) | Good improvement for 6 months.<br>Pleural effusions cleared.<br>Ascites for 3/12 | 11½ months from Primolut. |
| (14) L. L— (44) | Moderately differentiated cystadenocarcinoma | 11.i.61. Laparotomy " ascites + + +. Left ovary removed " | 26.i.61. 100 millicuries $198_{Au}$<br>6.vi.61. DXR | Metastases noted at laparotomy, " peritoneum covered with secondaries. Mesentery very much involved " | 14.vi.61. 6 × 250 mg. injections commenced | Good | Still alive and well. |
| (15) C. W— (55) | Poorly differentiated papillary adenocarcinoma | v.58. Total hysterectomy and bilateral salpino-oophorectomy | 27.v.58. DXR.<br>8.i.59. DXR to pelvis and gluteal region | 8.i.59. Hard mass encroaching upon lumen of rectum | 31.iii.59. 3 × 250 mg. injections.<br>21.iv.59 to 20.v.59. Primolut N. 10 mg. *t.d.s.* | Nil | 13½ months from surgery and DXR.<br>3 months from Primolut. |

| Case (Age) | Histology | Surgery | Radiotherapy | Recurrence (R) or Metastases (M) | Primolut | Improvement | Survival |
|---|---|---|---|---|---|---|---|
| (16) G. V— (44) | Well differentiated papillary adenocarcinoma | 24.x.56. Laparotomy | 15.xi.56. DXR | | 9.ix.57. 6×200 mg. Total 1,200 mg. | Nil | 13 months from surgery and DXR.<br>3 months from Primolut. |
| (17) L. A— (67) | Moderately differentiated adenocarcinoma | 4.vii.61. Laparotomy. Ascitic fluid—several pints | Nil | | 17.vii.61. 20×250 mg. injections (5,000 mg.) | Nil | 5 months from Primolut. |
| (18) T. B— (60) | Poorly differentiated cystadenocarcinoma | vi.1958. Bilateral salpingo-oophorectomy | 9.xi.59. DXR | x.1959. Abdomen and ascites (R) | ii.1960. Injections commenced | Nil | 20 months from surgery.<br>4 months from DXR.<br>1 month from Primolut. |
| (19) L. D— (47) | Papillary adenocarcinoma of both tubes | 3.xii.57. Total hysterectomy and bilateral salpingo-oophorectomy | 15.i.58. DXR | 3.xii.57. Number of soft nodules scattered throughout the pelvis, omentum and intestines.<br>5.xi.58. P.R. Tangerine-sized mass in right sacro-iliac region | 25.ii.59. 3×250 mg. injections (750 mg.) | Nil | 18 months from surgery.<br>16½ months from DXR.<br>3 months from Primolut. |

### TABLE IV.— *Malignant Tumours of Uterus*

| Case (Age) | Histology | Surgery | Radiotherapy | Recurrence (R) or Metastases (M) | Primolut | Improvement | Survival |
|---|---|---|---|---|---|---|---|
| (20) K. W— (51) | Well differentiated leiomyosarcoma | 21.vi.60. Total hysterectomy and bilateral salpingo-oophorectomy | 4.vii.60 to 17.vii.60 DXR. Treatment discontinued | Mass in left pelvis. Pain | Weekly injections since ii.61. 52 × 250 mg. (13,000 mg). | Very good throughout. Mass smaller | Still alive and well. |
| (21) H. M— (69) | Well differentiated adenocarcinoma (1955) | | 7.ii.55 to 14.ii.55 Radium at Nottingham General Hospital | 9.viii.60. Mass at vaginal vault with ulceration (R) | 15 injections since 29.vii.61 (4,250 mg.) | Very good throughout. Considerable regression of tumour and closure of vesico-vaginal fistula | Still alive and well. |
| | Ditto (1959) | 24.vii.59. Pan hysterectomy at Nottingham | iv.61. Cobalt treatment at Mount Vernon Hospital | | | | |
| (22) W. P— (54) | Leiomyosarcoma (tubes and ovaries) | 13.i.61. Pan-hysterectomy | 2.ii.61 to 7.iii.61. DXR | | 7.iii.61. 18 × 250 mg. injections (4,500 mg.) | 3 months. Fair | 8 months from from Surgery. 6 months from Primolut. |
| (23) D. I— (42) | Adenocarcinoma | 13.ii.59. Dilatation and curettage<br>2.iv.59. Wertheim hysterectomy.<br>31.v.61. Lt. iliac colostomy | 5.iii.59. One insertion of radium | 10.v.61. Bulky mass fixed to anterior rectal wall (M) | vi.61. Injections commenced. 9 × 250 mg. (2,250 mg.) | Fair for 3 months | 11½ months from surgery. 8 months from Primolut. |
| (24) A. E— (70) | Leiomyosarcoma | Nil<br>3.vii.59. Emergency laparotomy, appendicectomy and drainage of Pouch of Douglas | 23.vi.59. One insertion of radium<br>25.vi.59 to 3.vii.59. DXR. (Treatment not completed) | 28.x.59. Ulcerated growth in vagina (R) | 28.x.59. Primolut Depot | None | 9 months from R/therapy. 5 months from Primolut. |

| Case (Age) | Histology | Surgery | Radiotherapy | Recurrence (R) or Metastases (M) | Primolut | Improvement | Survival |
|---|---|---|---|---|---|---|---|
| (25) B. S— (53) | Poorly differentiated adenocarcinoma | Nil | 22.ix.60. Implantation of radium needles to anterior vaginal wall. Radium tube to uterus. Cobalt therapy. All at Churchill Hospital, Oxford | 30.viii.60. Metastasis in vaginal wall | 27.vi.61. Injections 6 × 250 mg. (1,500 mg.) | None | 12½ months from R/therapy. 3 months from Primolut. |
| (26) A. M— (61) | Cervical squamous epithelioma | Nil | 22.v.47 to 26.vi.47. DXR. 28.vi.47. One insertion of radium | 22.iii.61. Hard mass in suprapubic region with hard mass felt on P.V. and P.R. which is filling pelvis and encroaching upon lumen of rectum | Weekly injections since iii.1961. 43 × 250 mg. (10,750 mg.) | Good throughout | Still alive and well. |
| (27) E. B— (71) | Squamous cell carcinoma | Nil | ii.58. Radium treatment at Royal South Hants Hospital, Southampton. 16.vi.58. DXR to pelvis at Northampton | Extensive pelvic mass with ulcerated growth in vaginal vault | 22.x.58. Primolut Depot commenced | None | 13 months from R/therapy. 4 months from Primolut. |

Twenty-seven patients with tumours of the breast, ovary and uterus, in very advanced stages, in which other methods of treatment—surgery, radiotherapy, oestrogens or androgens or chemotherapy—have failed or have lost their effectiveness have been treated.

Of the 27 cases here reviewed there were 9 cases of carcinoma of breast, 10 cases of carcinoma of the ovary and 8 cases of malignant tumours of the uterus (2 cervical cases). The dosage was 250 mg. once a week ; in 2 cases the compound was given in tablet form—Norethisterone—30 mg. daily.

The Primolut Depot, 17α-hydroxyprogesterone capronate, has not in this series caused any untoward side effects. There were no reactions at the site of the injections.

Fifteen patients have shown subjective improvement, with increased appetite and a sense of well being. In some, objective signs of improvement were noted.

Table I shows the response to progesterone treatment assessed on the basis of clinical and radiological findings, and the statements of the patients' own general practitioners, and patients themselves concerning their objective and subjective improvement.

TABLE I.—*Cases Reviewed*—27

| Malignant tumours of: | Number of patients | Clinical response | | |
|---|---|---|---|---|
| | | Good | Fair | None |
| Breast | 9 | 3 | 2 | 4 |
| Ovary | 10 | 4 | 1 | 5 |
| Uterus | 8 | 3 | 2 | 3 |

Details of age, histology, previous surgery, radiotherapy, hormonetherapy or chemotherapy, the dosage of progesterone administered and response are set out in Tables II, III and IV.

In the series of 9 cases of advanced carcinoma of breast, the outstanding success of treatment with progesterone was that of Case 2 with skin recurrences in the pectoral region and abdominal metastases, and subsequently a tumour mass in the opposite breast which remains quiescent. This patient is doing well and has been on progesterone for over 3½ years, with a total dose of nearly 60,000 mg. Not only were there no untoward side effects but the patient's well-being has been maintained throughout. The suggestion made to the patient to discontinue progesterone for a while in order to enable an assessment of its real subjective value met with a flat refusal because of the experience gained from the occasional omission or delay of the weekly dose which had brought a deterioration in the patient's general condition, unduly easy fatigue and a sensation which can only be described as that of deprivation.

Similar statements were volunteered by 2 other patients. One with leiomyosarcoma of the uterus (Case 20) who has benefited both subjectively and objectively (reduction in size of palpable mass in pelvis, abolition of pain and improvement of general condition), and a patient with an ovarian tumour (Case 13) with ascites and bilateral pleural effusions, in whom progesterone was thought to be responsible for the drying up of the pleural effusion altogether until the time of death, and had at first also prolonged considerably the period between paracenteses. The latter, however, had to be subsequently resumed. In this patient clinically the undoubted effect was short lived (3 months), but it must be added that this particular patient had received also, at the beginning of the series of progesterone injections, nitrogen mustard (Thiotepa, $5 \times 15$ mg.).

In the other cases in whom the results were noted as " fair improvement " there was no doubt of the slight amelioration of the patients' condition. It must be stressed, however, that the dosage has been on the low side, and it seems that at least initially a dose of 500 mg. weekly is indicated.

The drying up of the ascites and pleural effusions after progesterone treatment was the outstanding feature in Case 13, and of ascites in Cases 10 and 17.

Similar results were observed by Kelley and Baker (1961) who used various preparations of progestational agents including aqueous suspension of progesterone in oil and 17 $\alpha$-hydroxyprogesterone capronate in weekly doses ranging from 150 to 1000 mg. Six out of 21 patients with endometrial carcinoma benefited (5 showed regression of pulmonary metastases), the remissions lasting from 9 months to $4\frac{1}{2}$ years. Three of these died ; in one the disease progressed and two enjoyed a remission on continual progesterone therapy.

Of the few results of treatment with free progesterone used, before synthetic preparations allowing the administration of high dosages were available, the following are worth recording. While Hertz, Cromer, Young and Westfall (1951) noticed visible and palpable regression of carcinoma of cervix uteri in 11 out of 17 patients treated with free progesterone, Gordon, Horwitt Segaloff, Murison and Schlosser (1952) noted improvement only in 2 out of 20 patients with breast cancer treated with free progesterone, and Volk, Escher, Huseby, Tyler and Cheda (1960) had no success with oral progesterone (2000 mg. daily) in 29 post menopausal women with advanced breast carcinoma. The only case of carcinoma of breast (Geller, Volk and Lewin, 1961) treated with synthetic progesterone was that of a male patient whose secondary bone deposits showed recalcification after treatment with 17 $\alpha$-hydroxyprogesterone—250 mg. daily.

### DISCUSSION

No impressive results could be expected in the group of patients here described, and the response noted as fair was that which is encountered in a proportion of cases treated with oestrogens or androgens. When, however, allowance is made for the inadequate amounts of progesterone actually taken because of the terminal stage at which the treatment was instituted and the interesting observations of reduction in size of tumour, drying up of effusions and subjective improvements produced, the value of progestins in treatment of some tumours appears to warrant a further study of their effects in malignant disease. As a sound approach to hormone therapy must be based not only on research in the endocrinological and clinical fields, but have a background of ideas concerning the mechanism of action of a new compound, i.e. a rationale for its application, a discussion of the latter seems desirable. For this reason the effects of progestins on the humoral and tissue levels will be briefly discussed.

The mechanism of action of progestins is not clear. It is worth mentioning that Jenkins (1961), who has described the effects of progesterone in cyclical ascites, is of the opinion that the electrolyte excretion plays an important role in this respect, progesterone acting as an aldosterone antagonist. This is probably borne out by the effects on ascites and pleural effusion in the cases of ovarian tumour.

It is not yet clear whether intermediate metabolites come into action, and it is not certain whether all progestins have an inhibitory effect on gonadotrophin secretion. It is assumed that Primolut Depot has very little direct effect on the pituitary. Other progesterones may have different mechanisms of action. The group of Edinburgh workers (Douglas, Loraine and Strong, 1960) found that 19-norethistosterone oenanthate in 12 cases of mammary carcinoma had little effect, only one case showing improvement. However, there are differences in the metabolism and physiological effects of the two progesterones, and this aspect awaits further investigation. In Table V are set out data concerning the metabolism of a few progestins and their side effects.

TABLE V.—*Effects of some Progestins*

| | Free progesterone | 19-Norethisterone | 17 α-hydroxy-progesterone capronate (Primolut Depot) | Norethynodrel (Enavid) |
|---|---|---|---|---|
| Pituitary gonado-trophins | Inhibition | Reduced, but not abolished | Weak antigonado-trophin | Reduced but not suppressed. |
| Oestrogen excretion | Increased | Increased, variable oestrinisation or little change | Not affected, no effect on menstrual cycle | Increased. |
| Androgenic ? or anabolic effects | ? | ? | No virilising effects in animals. Possible anabolic effect | — |
| Total 17-oxo-steroid and hydroxycortico-steroid excretion | Increase in pregnanendiol | Not reduced. Not depressed. ACTH increased | Not converted into progesterone, androgens or corticosteroids. Decrease in urinary pregnanendiol. ACTH not increased | Decreased. Reduced pregnanendiol. |
| Electrolytes Ca, K, Na, N, Cl. | Aldosterone antagonist | No change (Loraine). Slight retention of Na (Jenkins) | Increased excretion of Na. Aldosterone antagonist | — |

Titres of follicle-stimulating hormone in one patient measured by Kelley and Baker (1961) showed no suppression of the pituitary during treatment. Sherman and Woolf (1959) noted a drop in elevated levels of luteinizing hormone in 4 patients with carcinoma of the endometrium who received 17-hydroxyprogesterone capronate for six weeks. As these authors do not state whether there has been tumour regression in their cases the question of an effect via the pituitary gland remains an open one.

Sherman and Woolf (1959) suggest that in cases of endometrial carcinoma an increased LH production exercises an influence on an abnormally high complement of hilar (Leydig or theca) cells which are kept in check during reproductive years by progesterone derived from successive corpora lutea. After menopause this restraint is no longer available and the ensuing excessive oestrogen production is followed by an endocrinal pattern leading through a phase of cystic adenomatous hyperplasia and anaplasia to carcinoma. By introducing into the body of a hormone which may act by the proper feed-back action on the pituitary the endocrine pattern, as suggested by these authors, might be interfered with either prophylactically or as a curative measure in established neoplasia.

Kistner (1958) who studied the effect of 17$\alpha$-hydroxyprogesterone in endometriosis suggests that the esterified steroid is transported intact to the tissues where it exerts its biologic activity, and there is slowly metabolized as it induces its action. Brown, Fotherby and Loraine (1960) in their study of the effects of 17$\alpha$-ethinyl-19-norethisterone on the urinary excretion of oestrogens, pregnanendiol and gonadotrophins during the menstrual cycle postulate that norethisterone exerts its effect by a direct action on the ovary rather than through the pituitary. It is generally assumed that oestrogens exert their influence on malignant cells directly, and little attention has been paid to histological changes produced in intercellular structures deeply affected both structurally and functionally by steroids. The importance of these changes cannot be over-rated.

Facts regarding histological changes by endocrine glands on connective tissue are very fragmentary. Important clues concerning the anatomical and functional state of tissues in hormone dependent organs (uterus, breast) come from observations of the physiological changes which these organs undergo during lactation, the menstrual cycle and in the menopause. The oedema of the stroma in the proliferative and secretory phases of the menstrual cycle, followed by the destruction and shedding of this structure with the menstruating endometrium (Zuckerman, 1949) is most interesting in this context.

Muller (1951) found that the effects of subcutaneously injected oestrogen on loose connective tissue in rats were different from those on the dense connective tissue. In the former oestrogen induced a rejuvenation process, as shown by the development of a fine reticular network and an increase of ground substance, while in the dense connective tissue it induced a maturation process with an increase in the denseness and number of collagen bundles.

Pliske (1953) found in guinea-pigs marked hypertrophy of epidermal cells and swelling of the collagen fibre bundles producing a nearly homogeneous condition in the tissue with interfibrous vacuoles containing degenerating fibroblast nuclei appearing regularly between the fibre bundles.

Jolles, Greening, Dun and Timms (1956) found in a series of rabbits' ears treated with local estrogen applications that the most marked changes were in the epidermis and the collagen of the dermis.

In the epidermis, contrary to the findings of Pliske (1953), no decrease in the number of layers of epidermal cells was noted ; an increase in these was found constantly in the experimental and control ears—in the latter due probably to oestrogen released into the circulation. In the dermis, swelling of the collagen fibres and an appearance of " homogeneity " of the dermis appeared to be characteristic. Examination of fibres by means of electron microscopy did not reveal changes in the amount of coating material noted in experiments in which a reversible " maturation " of collagen (characterized by a decrease due to dispersal or clearing of coating material of collagen fibrils) was induced by mechanical means such as stretching.

The " antifibromatogenic " (Lipschütz, 1950) effect of progesterone has been known for a long time, but the main indications for its use in the gynaecological field have been endometriosis, dysmenorrhoea and threatened habitual abortion.

The effects of progesterone on experimental tumour growth is that of acceleration on the one hand (Ehrlich tumours in rats)—latency period halved, followed by rapid growth, while on the other hand if the rats were pretreated with progesterone their survival was prolonged (Rio, 1957).

A compound such as synthetic progesterone which has the effect of a " decidual transformation " of the endometrial stroma may play an important role in the management of tumours in hormone-dependent organs. Changes in tumour stroma and mesenchymal tissues in general may affect not only the rate of spread but induce a regression of tumour when the prevailing conditions are rendered unfavourable. The physio-chemical state of intercellular tissue components, the degree of their imbibition, the state of the connective tissue ground substance in tumour and tumour bed have to be considered. It is relevant to recall the fact that oedema unfavourably influences tumour response in radiotherapy. Changes in tumour stroma and tumour bed brought about by ionizing radiation represent a relevant part of the beneficial reaction leading to the destruction of tumour and repair (Jolles and Koller, 1950; Jolles, 1953). Untimely and excessive fibrosis in irradiated tumours may on occasion jeopardize the outcome of treatment. Thus, a hormone which causes the disappearance of fibrosis in endometriosis could be used with advantage as a coadjuvant of other forms of therapy.

The key position of progestins in steroid metabolism renders this hormone interesting when one considers the advisability of an attack on many fronts at an early stage of malignant disease. The clinical observations made in the series of cases described are encouraging enough and it is hoped that further experience in less advanced stages of malignant disease will throw more light on this and related problems.

SUMMARY

Twenty-seven patients with advanced malignant tumours of the breast (9), ovary (10) and uterus (8) were treated with a progesterone steroid $17\alpha$-hydroxy-progesterone capronate. A good response was noted in 10 cases. A rationale of treatment and the possible mechanisms of action of this hormone are discussed.

I wish to thank my surgical and gynaecological colleagues for their co-operation and Mr. G. Raivid, M.P.S., of Pharmethicals (London) Ltd. for the supply of Primolut in the early stages of the investigation.

### REFERENCES

Brown, J. B., Fotherby, K. and Loraine, J. A.—(1960) *Proc. Roy. Soc. Med.*, **53**, 431.
Douglas, M., Loraine, J. A. and Strong, J. A.—(1960) *Ibid.*, **53**, 427.
Geller, J., Volk, H. and Lewin, M. (1961) *Cancer Chemother. Rep.* **14**, 77.
Gordon, D., Horwitt, B. N., Segaloff, A., Murison, P. J. and Schlosser, J. V. (1952) *Cancer*, **5**, 275.
Hertz, R., Cromer, Y., Young, J. and Westfall, B. (1951) *J. nat. Cancer Inst.*, **8**, 123.
Jenkins, J. S.—(1961) *Brit. med. J.*, ii, 861.
Jolles, B.—(1953) ' X-ray Sieve Therapy in Cancer. " A connective Tissue Problem " '. London (H. K. Lewis).
*Idem*, Greening, S. G., Dun, G. B. and Timms, P.—(1956) *Nature, Lond.*, **178**, 148.
*Idem* and Koller, P. C.—(1950) *Brit. J. Cancer*, **4**, 77.
Junkmann, K.—(1954) *Arch. exp. Path. Pharmak.*, **223**, 244.
Kelley, M. and Baker, W. H.—(1961) *New Engl. J. Med.*, **264**, 216.
Kistner, R. W.—(1958) *Amer. J. Obstet. Gynec.*, **75**, 264.

LIPSCHÜTZ, A.—(1950) 'Steroid Hormones and Tumours'. Baltimore (Williams and Wilkins Co.).
MULLER, T.—(1951) *Anat. Rec.*, **111**, 355.
PLISKE, E. C.—(1953) *Ibid.*, **115**, 673.
RIO, F.—(1957) *Arch. ital. Patol. clin. Tumori*, **1**, 569.
SHERMAN, A. I. AND WOOLF, R. B.—(1959) *Amer. J. Obstet. Gynec.*, **77**, 233.
STOLL, B. A.—(1961) *Cancer Chemother. Rep.*, **14**, 83.
SUCHOWSKY, G. K. AND JUNKMANN, K.—(1961) *Endocrinology*, **68**, 341.
VOLK, H., ESCHER, G. C., HUSEBY, R. A., TYLER, F. H. AND CHEDA, J. (1960) *Cancer*, **13**, 757.
ZUCKERMAN, S.—(1949) *Lancet* i, 1031.


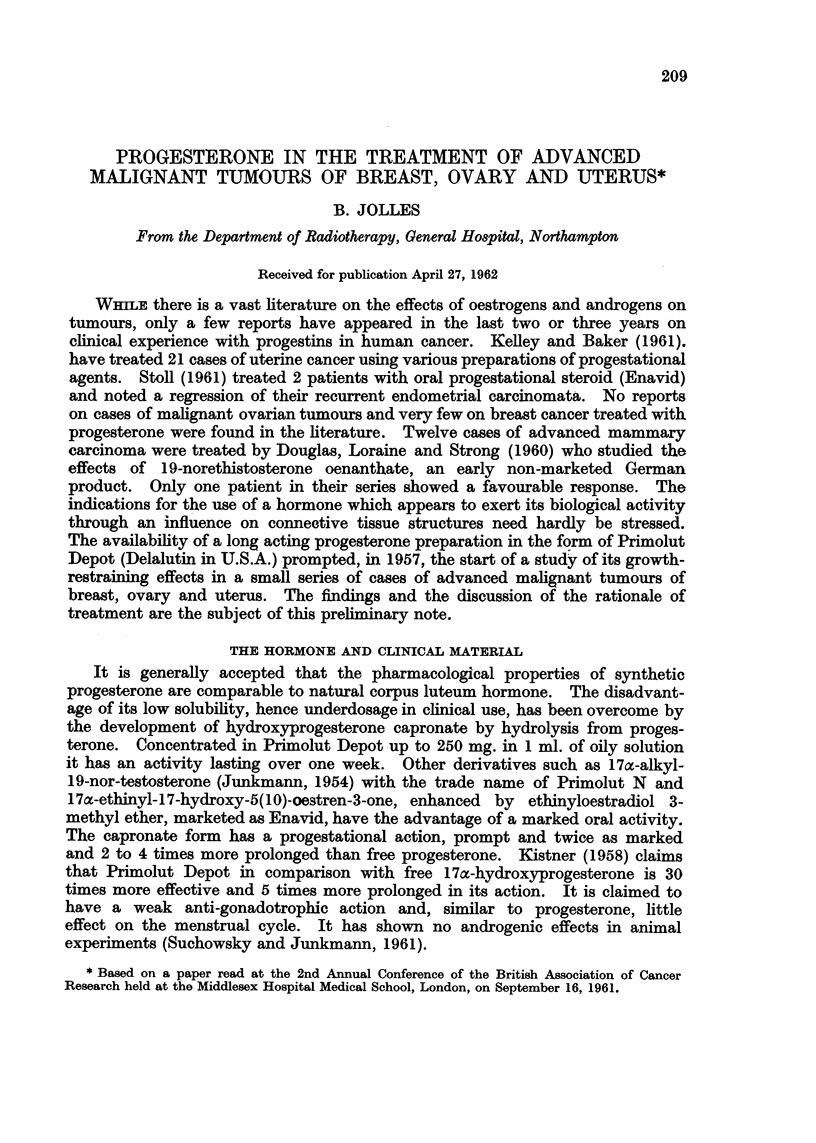

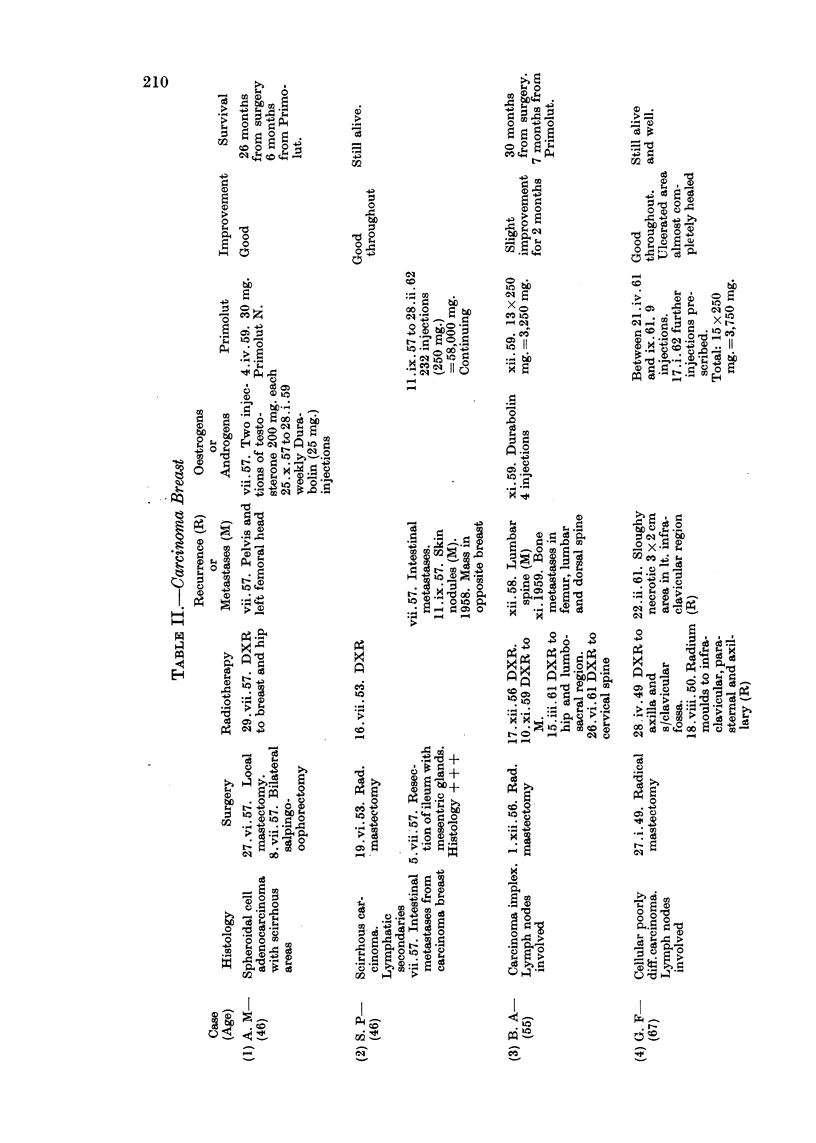

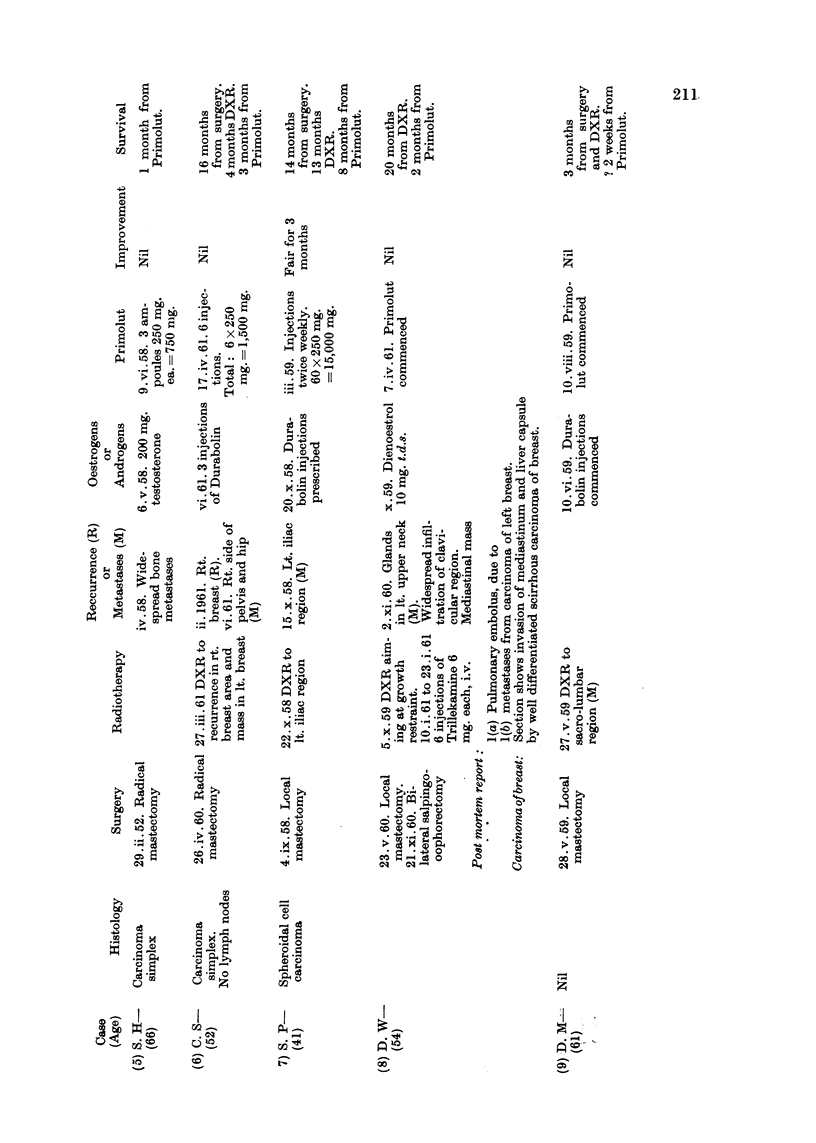

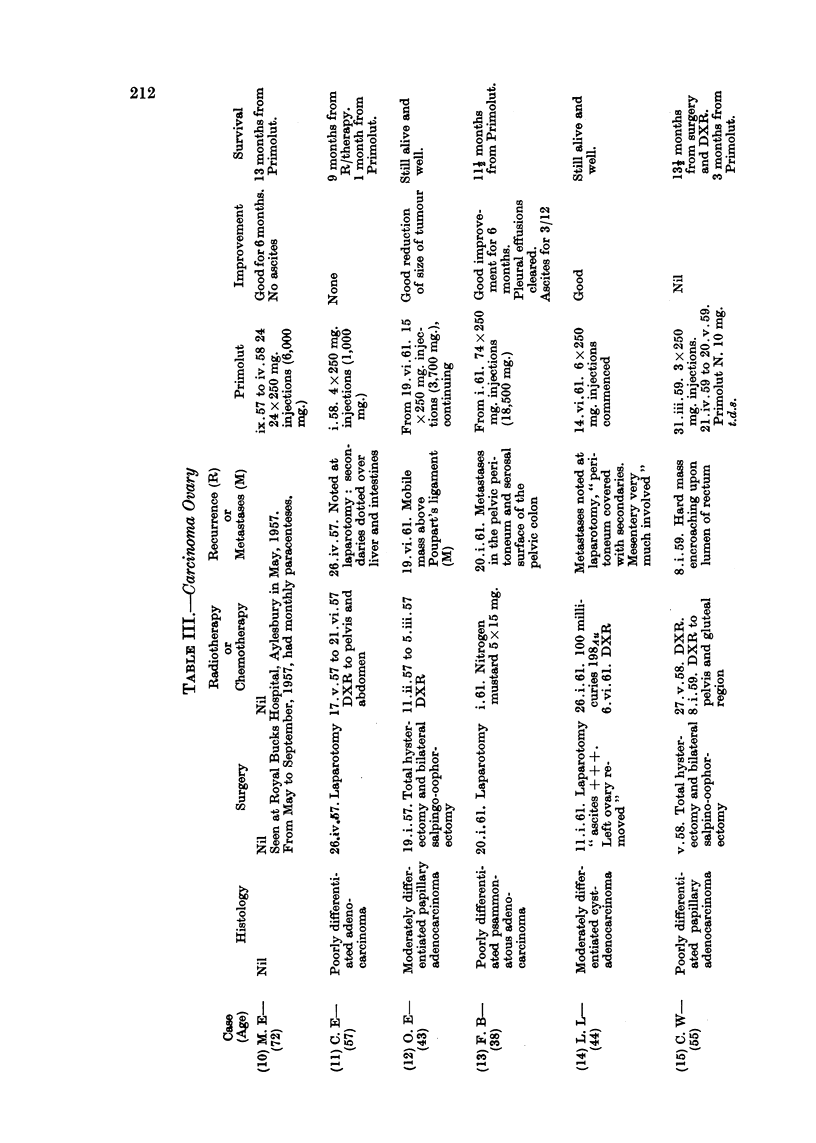

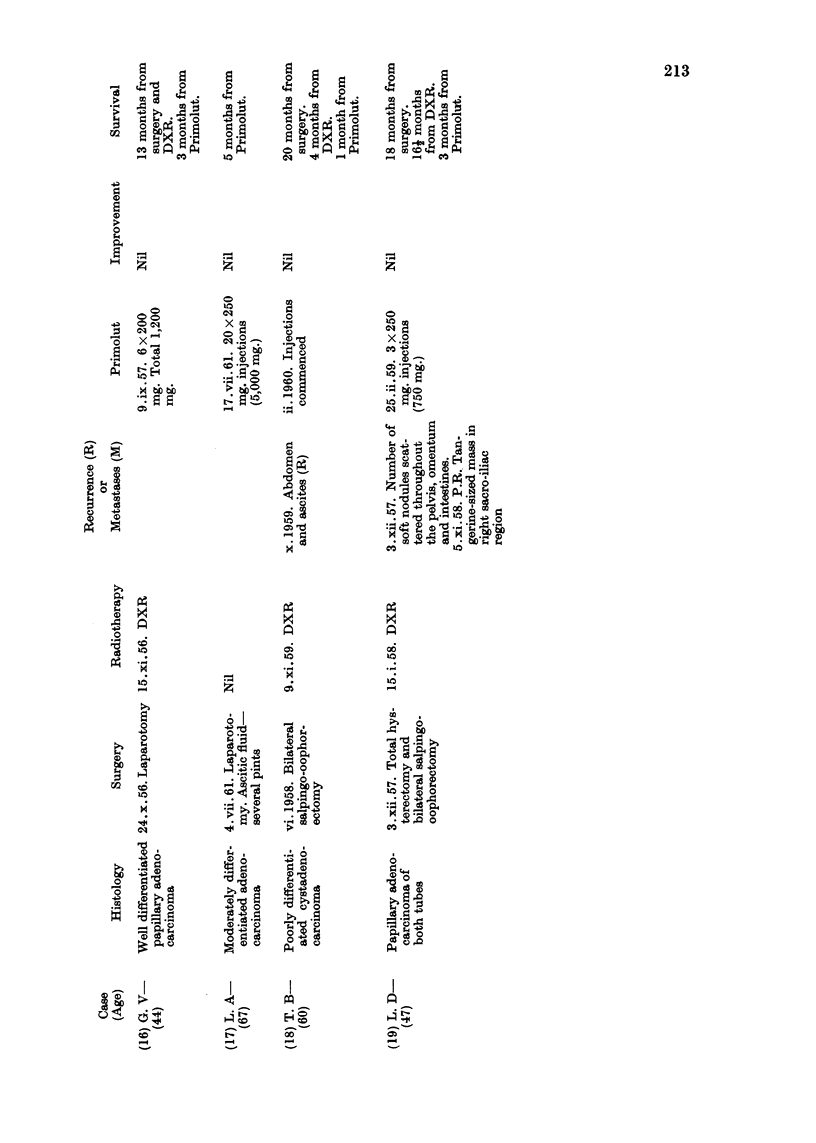

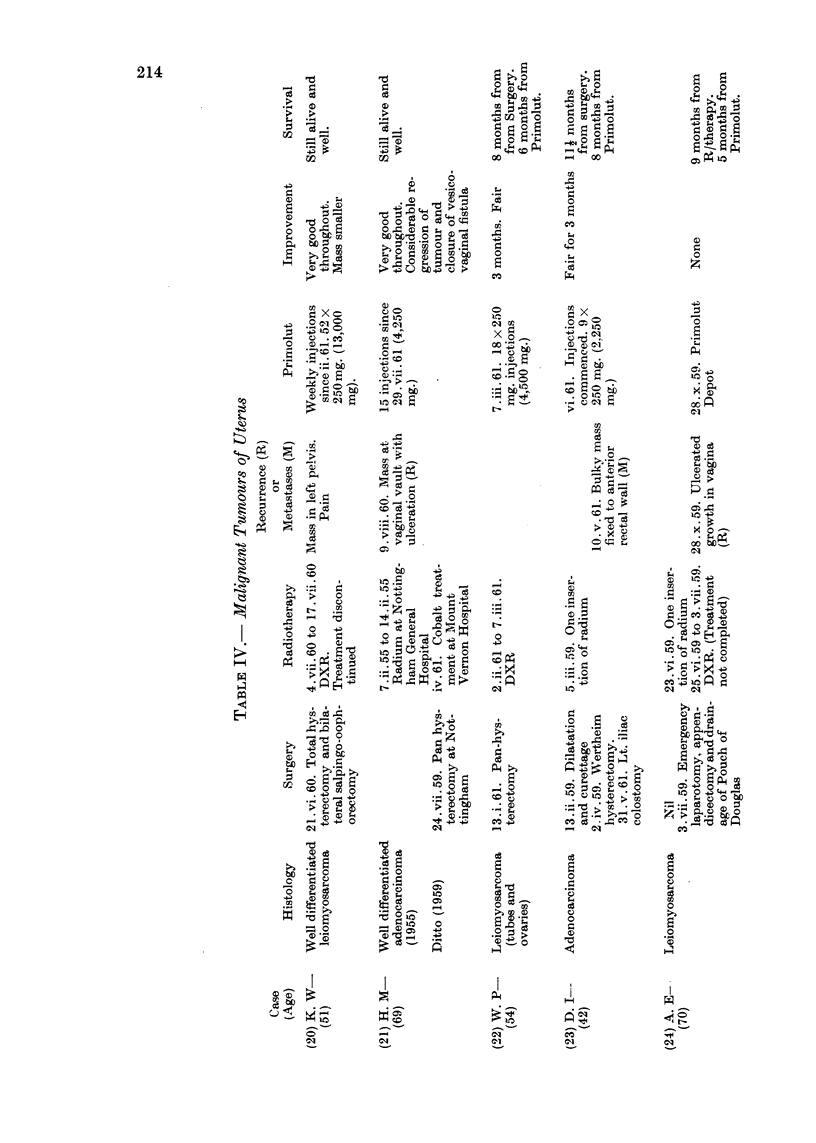

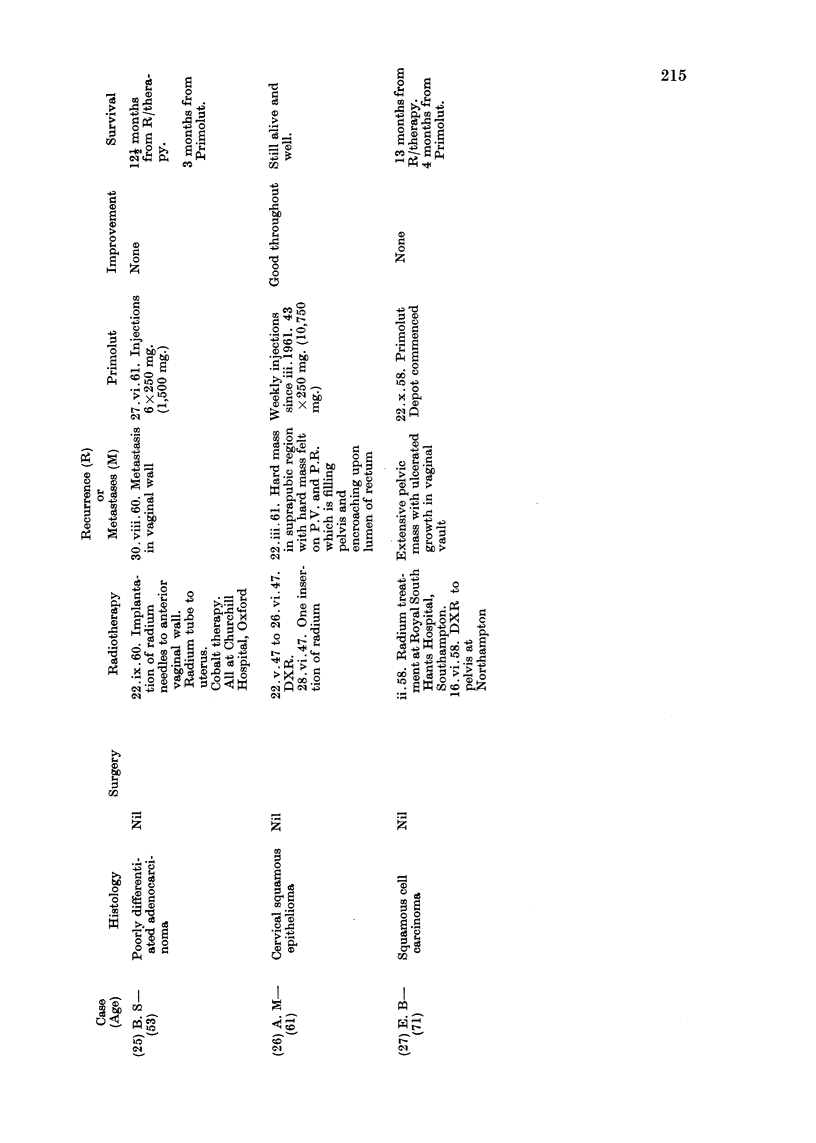

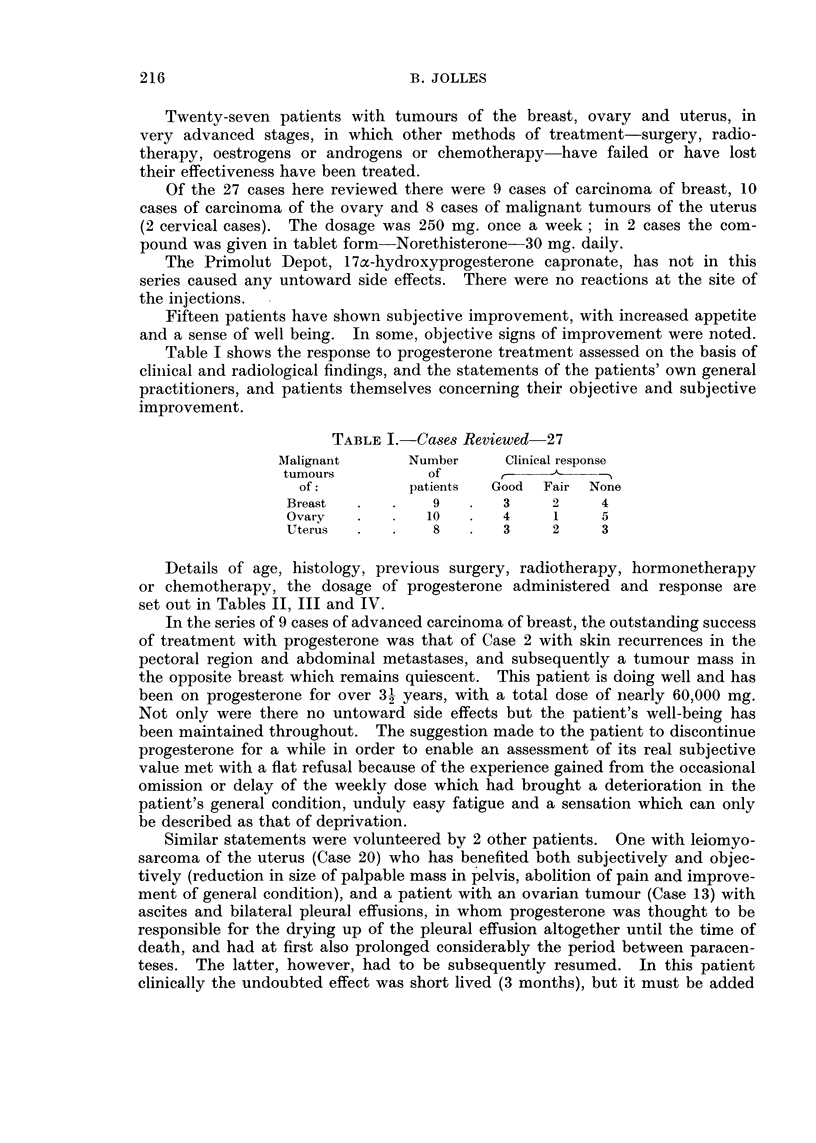

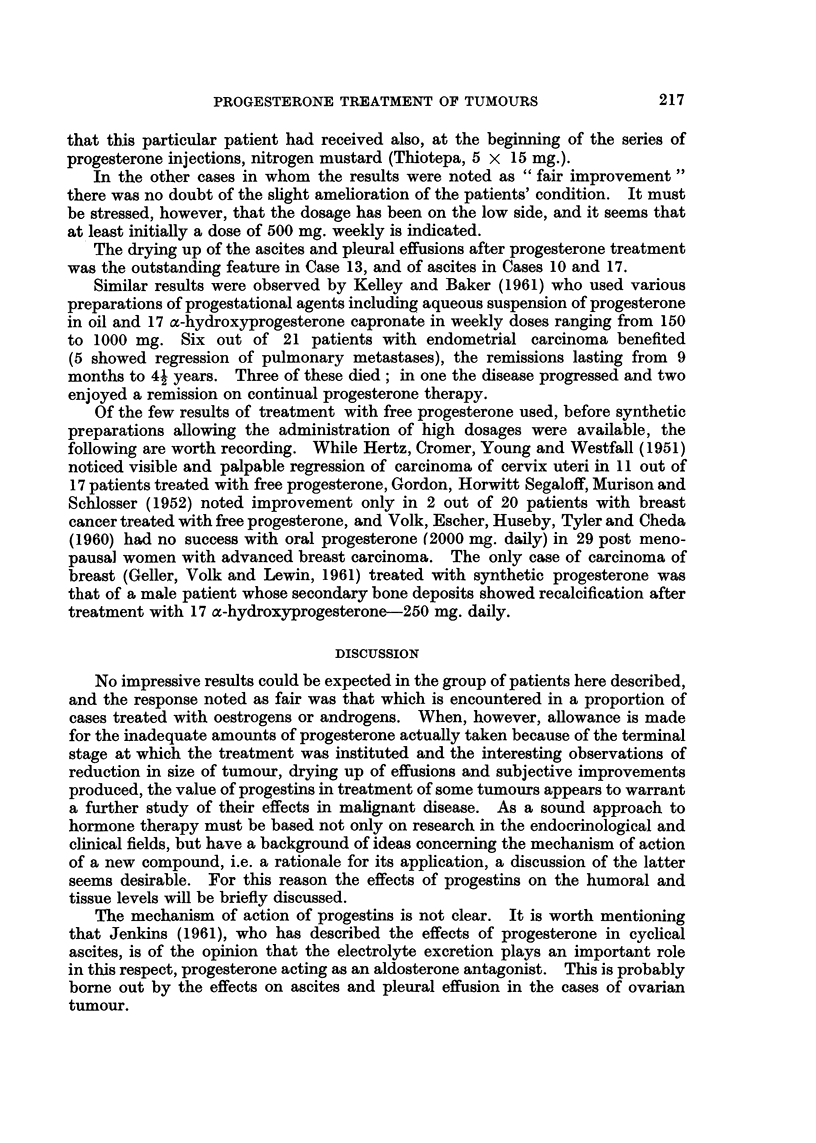

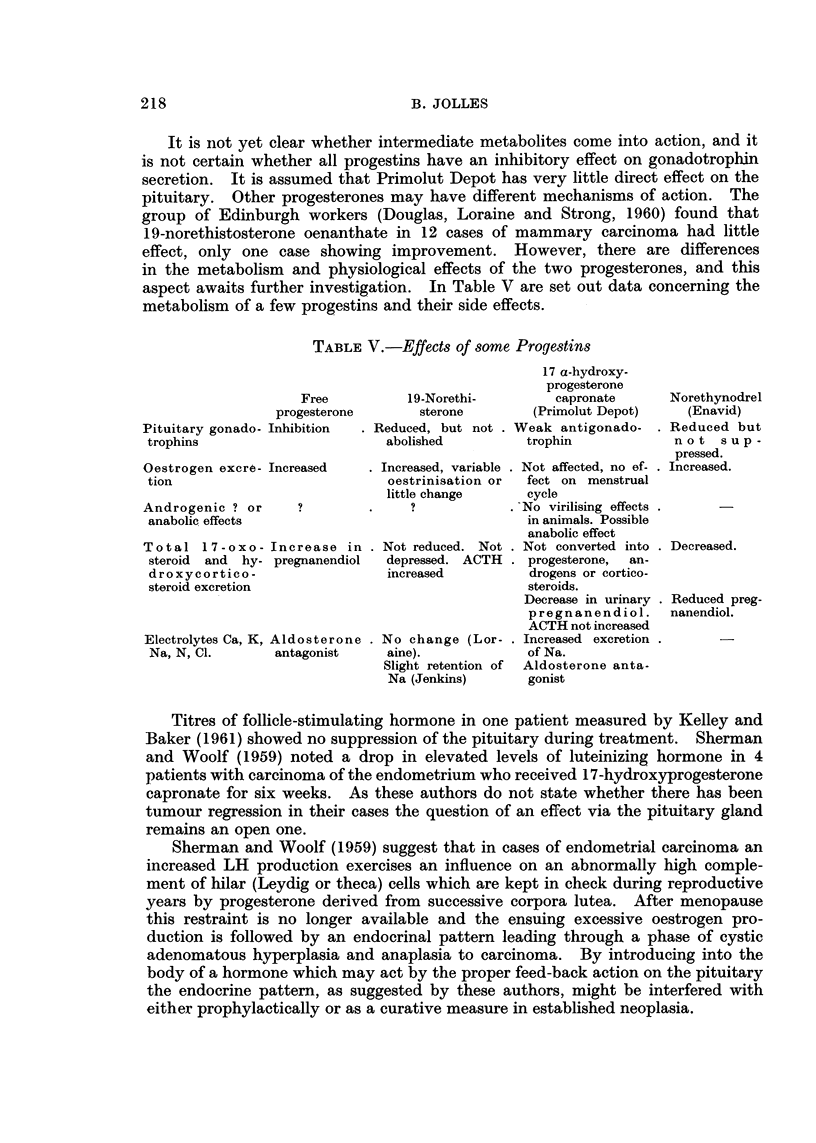

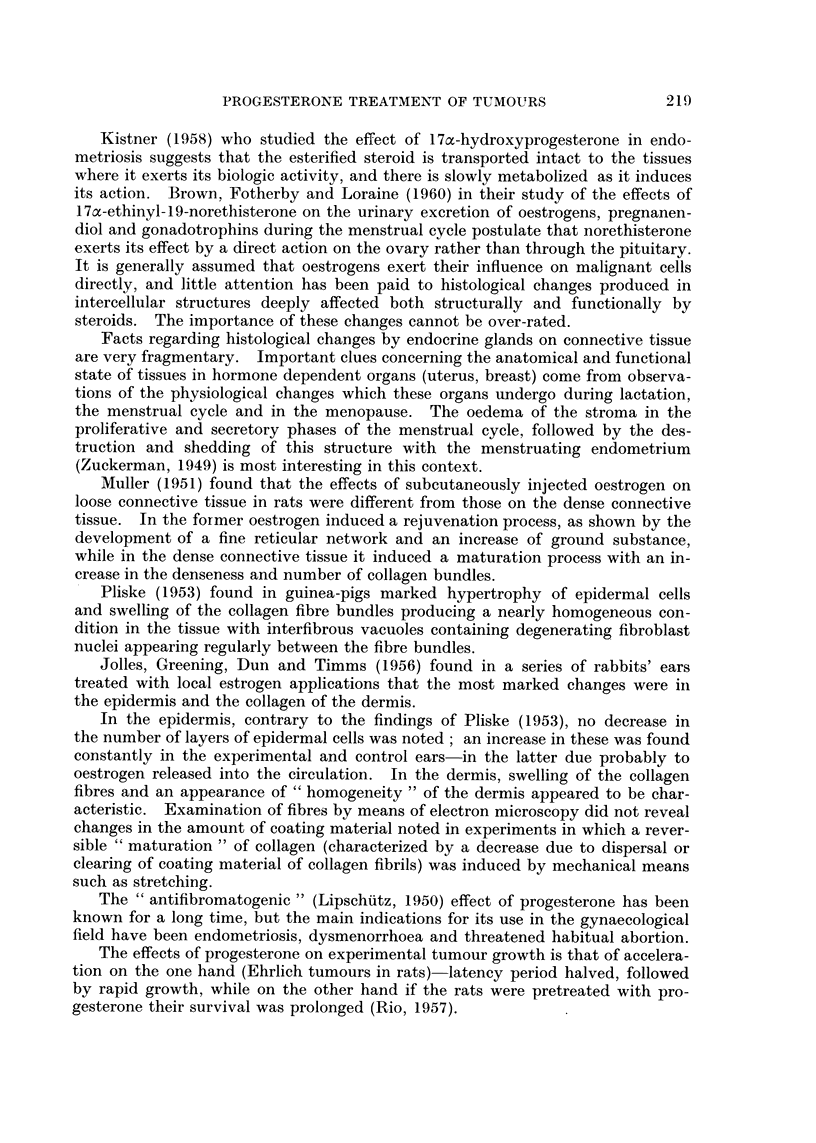

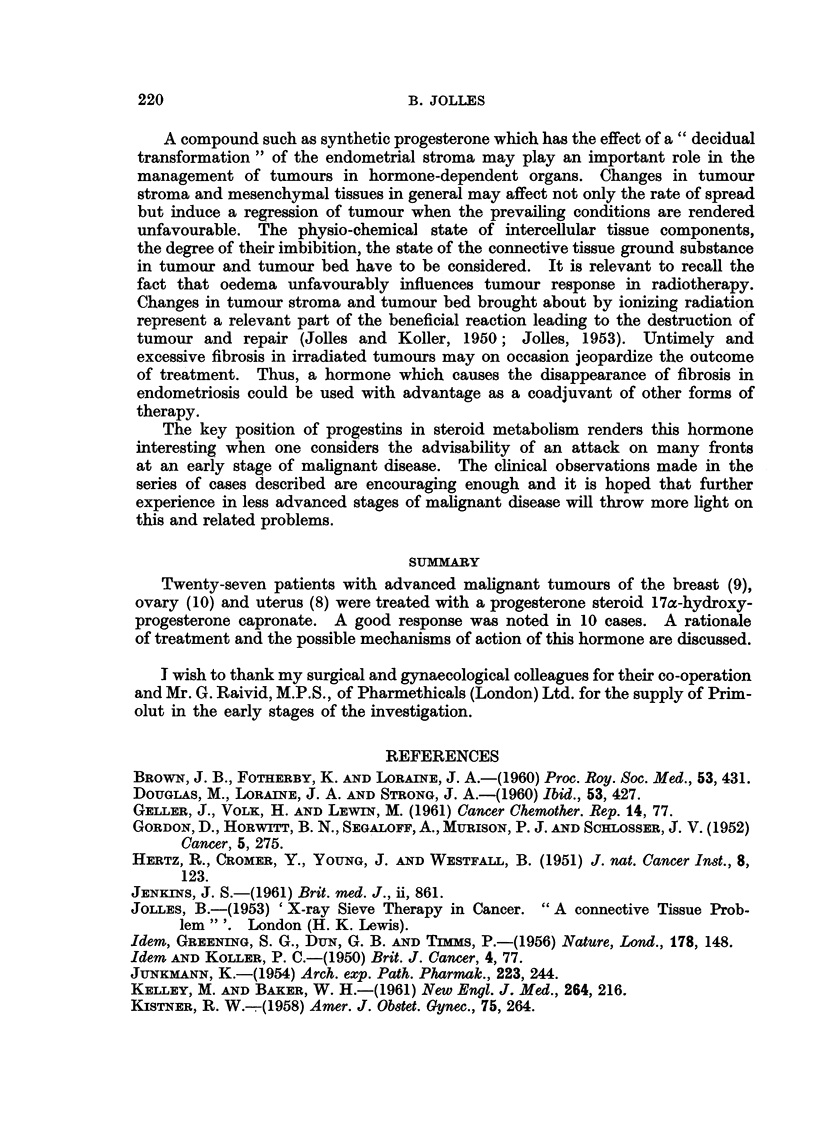

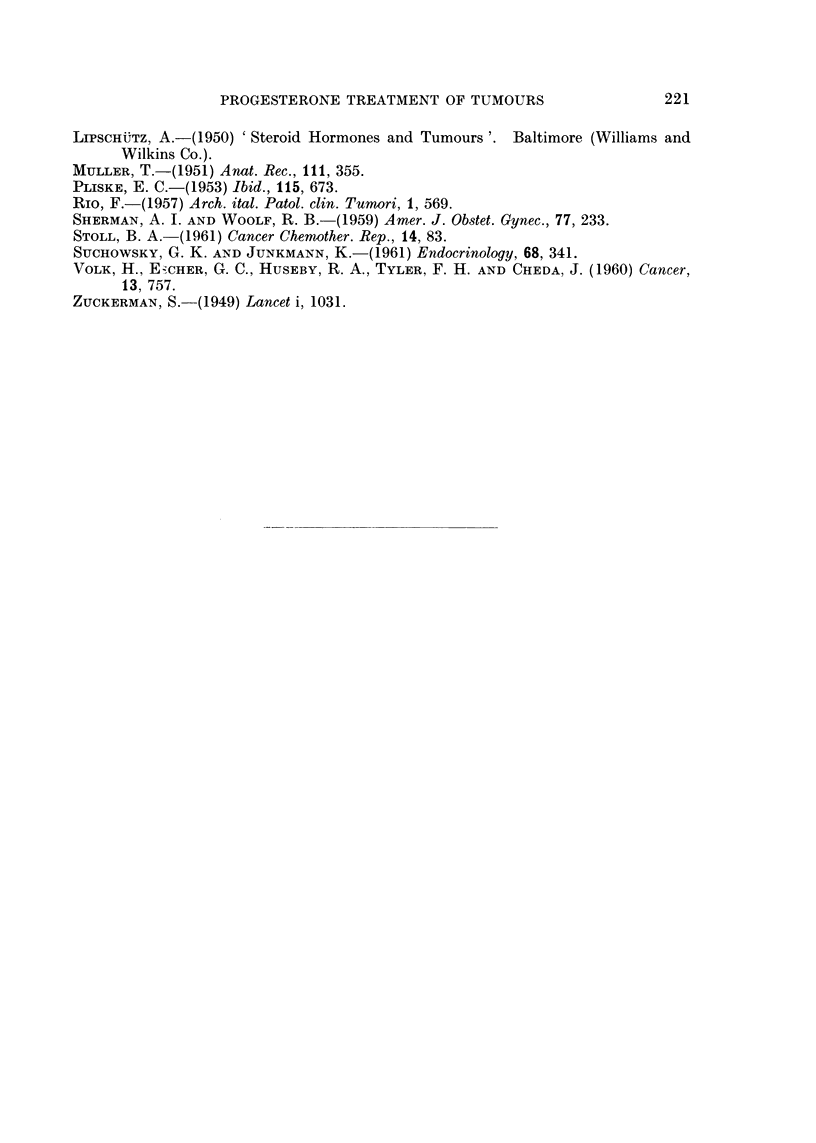


## References

[OCR_01247] BROWN J. B., FOTHERBY K., LORAINE J. A. (1960). The effect of 17alpha-ethinyl-19-nortestosterone (norethisterone) on the urinary excretion of oestrogens, pregnanediol and gonadotrophins during the menstrual cycle.. Proc R Soc Med.

[OCR_01248] DOUGLAS M., LORAINE J. A., STRONG J. A. (1960). Studies with 19-norethisterone oenanthate in mammary carcinoma.. Proc R Soc Med.

[OCR_01250] GELLER J., VOLK H., LEWIN M. (1961). Objective remission of metastatic breast carcinoma in a male who recieved 17-alpha hydroxy progesterone caproate (delalutin).. Cancer Chemother Rep.

[OCR_01252] GORDON D., HORWITT B. N., SEGALOFF A., MURISON P. J., SCHLOSSER J. V. (1952). Hormonal therapy in cancer of the breast. III. Effect of progesterone on clinical course and hormonal excretion.. Cancer.

[OCR_01267] JOLLES B., GREENING S. G., DUN G. B. (1956). Experimental maturation or ageing of collagen in rabbits' ears.. Nature.

[OCR_01269] JUNKMANN K. (1954). Uber protrahiert wirksame Gestagene.. Naunyn Schmiedebergs Arch Exp Pathol Pharmakol.

[OCR_01271] KELLEY R. M., BAKER W. H. (1961). Progestational agents in the treatment of carcinoma of the endometrium.. N Engl J Med.

[OCR_01272] KISTNER R. W. (1958). The use of newer progestins in the treatment of endometriosis.. Am J Obstet Gynecol.

[OCR_01280] MULLER T. (1951). The effect of estrogen on the loose connective tissue of the albino rat.. Anat Rec.

[OCR_01281] PLISKE E. C. (1953). Histologic changes in the skin of the female guinea pig following percutaneous application of estrogen.. Anat Rec.

[OCR_01285] SHERMAN A. I., WOOLF R. B. (1959). An endocrine basis for endometrial carcinoma.. Am J Obstet Gynecol.

[OCR_01286] STOLL B. A. (1961). A new progeshational steroid in the therapy of endometrial carcinoma--a preliminary report.. Cancer Chemother Rep.

